# Evaluation of the Antimicrobial Activity of Endophytic Bacterial Populations From Chinese Traditional Medicinal Plant Licorice and Characterization of the Bioactive Secondary Metabolites Produced by *Bacillus atrophaeus* Against *Verticillium dahliae*

**DOI:** 10.3389/fmicb.2018.00924

**Published:** 2018-05-09

**Authors:** Osama A. A. Mohamad, Li Li, Jin-Biao Ma, Shaimaa Hatab, Lin Xu, Jian-Wei Guo, Bakhtiyor A. Rasulov, Yong-Hong Liu, Brian P. Hedlund, Wen-Jun Li

**Affiliations:** ^1^Key Laboratory of Biogeography and Bioresource in Arid Land, Xinjiang Institute of Ecology and Geography, Chinese Academy of Sciences, Urumqi, China; ^2^Environmental Science Department, Institute of Environmental Studies, Arish University, El-Arish, Egypt; ^3^School of Life Sciences, University of Nevada, Las Vegas, Las Vegas, NV, United States; ^4^Department of Food Science and Technology, College of Environmental Agricultural Sciences, Arish University, El-Arish, Egypt; ^5^Key Laboratory of Hexi Corridor Resources Utilization, Hexi University, Zhangye, China; ^6^Key Laboratory of Crops with High Quality and Efficient Cultivation and Security Control, Yunnan Higher Education Institutions, Honghe University, Mengzi, China; ^7^Institute of Genetics and Plant Experimental Biology, Uzbekistan Academy of Sciences, Tashkent, Uzbekistan; ^8^State Key Laboratory of Biocontrol and Guangdong Provincial Key Laboratory of Plant Resources, School of Life Sciences, Sun Yat-sen University, Guangzhou, China

**Keywords:** medicinal plants, endophytes, environmental microbiology, biological control, *Verticillium dahliae*, *Bacillus atrophaeus*, Licorice

## Abstract

Endophytic bacteria associated with medicinal plants possess unique strategies that enhance growth and suvival of host plants, many of which are mediated by distinctive secondary metabolites. These bacteria and their secondary metabolites are important subjects for both basic and applied research aimed at sustainable agriculture. In the present study, 114 endophytic strains isolated from the wild ethnomedicinal plant *Glycyrrhiza uralensis* (licorice) were screened for their *in vitro* antimicrobial activities against common fungal pathogens of tomato (*Fusarium oxysporum* f. sp., *Fulvia fulva*, *Alternaria solani*), cotton (*Fusarium oxysporum* f. sp. Vesinfectum, *Verticillium dahliae*), pomegranite (*Ceratocystis fimbriata*), *Cymbidinium* (*Colletotrichum gloeosporioides*), and Tsao-ko (*Pestalotiopsis microspora* and *Fusarium graminearum*) and the common bacteria *Staphylococcus aureus*, *Bacillus cereus*, *Salmonella enteritidis*, and *Escherichia coli*. Several *Bacillus* strains, particularly *Bacillus atrophaeus* and *Bacillus mojavensis*, had a broad spectrum of antifungal and antibacterial activity. A total of 16 strains, selected based on broad antimicrobial activity, were shown to contain at least one putative secondary metabolite-encoding gene (i.e., polyketide synthase or non-ribosomal peptide synthetase) and/or one lytic enzyme (i.e., protease, cellulase, lipase, chitinase), which may be important mediators of antagonistic activity against pathogens. Five strains, representing *Bacillus atrophaeus* and *Bacillus mojavensis*, were selected for plant growth chamber experiments based on strong *in vitro* antifungal activities. All five strains significantly reduced disease severity in *Arabidopsis thaliana* plants challenged with *V. dahlia* infection. Gas-chromatography/mass-spectrometry analysis of cell-free extracts of *Bacillus atrophaeus* strain XEGI50 showed that at least 13 compounds were produced only during co-cultivation with *V. dahlia*, including putative compounds known to have antimicrobial activity, such as 1,2-benzenedicarboxylic acid, bis (2-methylpropyl) ester; 9,12-octadecadienoic acid (Z,Z)-, methyl ester; 9-octadecenoic acid, methyl ester, (E)-; and decanedioic acid, bis(2-ethylhexyl) ester. To our knowledge, this study is the first to report that bacteria isolated from *G. uralensis* have biocontrol abilities. Our findings provide new insights into the antimicrobial activities of natural endophytes, particularly *B. atrophaeus*, and suggest this species may a promising candidate as a biocontrol agent to confer resistance to *Verticillium* wilt disease and other phytopathogens in cotton and other crops.

## Introduction

Fungal disease is the main threat to both crop yields and global food security (Fisher et al., 2012). Vascular wilts are devastating plant diseases that cause major losses to food crops and destroy natural ecosystems (Yadeta and Thomma, 2013). Two major genera of pathogenic fungi, *Fusarium* and *Verticillium*, enter their host plants through the roots or are transmitted by beetles and cause vascular wilts. Both are characterized by a wide host range (Juzwik et al., 2008; Harwood et al., 2011). The signs of *Verticillium* wilts are similar to those of *Fusarium*, starting with yellowing of the older leaves, followed by chlorosis and necrosis. As a result, vascular discoloration and stunting may be visible (Ting, 2014). In China, approximately 3 million hectares of cotton crops are affected by *Verticillium* wilt, accounting for an annual loss of yield of 10–30% (Bibi et al., 2013). In addition, no fungicides are registered for controlling *Verticillium* wilt disease in cotton (Göre et al., 2009). Xinjiang Province, located in the northwest of China, produces 11% of the global cotton fiber yield and is disproportionately harmed by *Verticillium* wilt disease (Peng et al., 2016).

Controlling vascular wilt pathogens can be challenging due to the fact that there are no efficient approaches to treat infected plants. Chemical fungicides are costly, inefficient, and have adverse impacts on the environment and human health (Qing et al., 2015). Increasing concern about the environmental and human health impacts of traditional fungicides has spawned intense interest in the development of safer alternatives. Biological control, an eco-friendly alternative, is mediated by microbial antagonists that possess unique traits that enable them to inhibit the growth of fungal pathogens (Walker et al., 1998; Chernin and Chet, 2002; Berg and Hallmann, 2006). Several mechanisms are responsible for these antagonistic activities, including inhibition of pathogen growth via antibiotics, toxins, surface-active compounds (antibiosis), and extracellular digestive enzymes such as proteases, cellulases and chitinases (Chernin and Chet, 2002; de Souza et al., 2003). However, several studies of biocontrol agents have reported dissimilarities between the antagonistic effects *in vitro* and the corresponding *in situ* efficacy (Berg et al., 2000). Therefore, additional research is needed to better understand the basis by which plant-associated bacteria suppress fungal diseases *in vivo* (Santhanam et al., 2014), not only for basic science purposes, but also for the development of improved biocontrol approaches to support sustainable eco-friendly crop production (Weller, 1988; Sturz et al., 2000).

Endophytic bacteria have the capability to systematically colonize plant tissues and establish a symbiotic relationship with the host, which makes them highly efficient biocontrol agents (Bakker et al., 2013). Several reports have investigated bacterial endophytes as possible biocontrol agents against diverse pathogenic fungi (Lacava et al., 2007; Erdogan and Benlioglu, 2010; Egamberdieva et al., 2017a,b). Recently, several studies have described bacterial endophytes with biological control activity on a number of crops as potential sources of antimicrobial metabolites. The host plants in these studies have included *Solanum trilobatum* (Bhuvaneswari et al., 2013), *Nicotiana attenuata* (Santhanam et al., 2014), *Solanum trilobatum melongena*, and *Solanum torvum* (Achari and Ramesh, 2014).

In view of the importance of endophytes to plant health, and increased focus on traditional herbal remedies as alternatives to synthetic pharmaseuticals, recent studies have begun to probe the importance of endophytic bacteria to medicinal plants, particularly those growing in unusual or stressed environments (Strobel and Daisy, 2003; Vieira et al., 2011; Liu Y.-H. et al., 2016; Egamberdieva et al., 2017a; Liu et al., 2017). Many of these studies have fingered the genus *Bacillus*, due to its widespread abundance in different plants, its broad-spectrum antimicrobial activities (Zheng et al., 2013; Jiang et al., 2015; Gao et al., 2017), and its ability to form endospores that are highly resistant to abiotic stresses such as UV light, desiccation, and extremes of pH, salinity, and temperature (Horikoshi, 2008).

Bacteria associated with medicinal plants have rarely been explored with regard to antagonistic activity against plant pathogens (Bakker et al., 2013; Bhuvaneswari et al., 2013; Egamberdieva et al., 2017a). Licorice (*Glycyrrhiza uralensis*) is a popular traditional Chinese medicine. Licorice contains bioactive compounds such as phenolics, flavonoids, triterpene saponins, and coumarins (Zhang and Ye, 2009). According to Chinese medicine theory, licorice has many important pharmacological activities, including antimicrobial and antiviral activity, histamine inhibition, anti-inflammatory activity, detoxification, and antioxidant and antitumor activities (Liao et al., 2012). Despite the economic interest and broad pharmacological effects of the popular medicinal usage of *G. uralensis*, very little research has been conducted on its endophytes or the potential for its endophytes to be used as biocontrol agents (Asl and Hosseinzadeh, 2008). Therefore, the objectives of the present study were: (1) to screen a diverse collection of endophytic bacteria (18 genera and 34 species) isolated from wild populations of *G. uralensis* for activity against a variety of pathogens *in vitro*; (2) to evaluate selected isolates for their biological control efficiency against the vascular wilt pathogen *V. dahliae in vivo*; and (3) to identify prevalent volatile organic compounds (VOCs) produced by endophytes only in the presence of *V. dahliae*, which are likely to be among the effectors of the antimicrobial properties.

## Materials and Methods

### Endophytic Bacteria and Culture Condition

A collection of 114 endophytes were previously isolated from wild populations of *G. uralensis* (Li et al., 2018) from three areas in Xinjiang province, representing 18 genera and 34 species. All isolates were submitted to NCBI GenBank under Accession Number (KY127308 – KY127422) and used in this study. Bacterial isolates were stored in 20% glycerol at the Key Laboratory of Biogeography and Bioresource in Arid Land, Xinjiang Institute of Ecology and Geography, Chinese Academy of Sciences under -20°C. Bacteria were routinely cultured on ISP_2_ growth medium, and incubated at 28 ± 2°C for 48–72 h.

### Antibacterial Activity

A modification of the agar disk diffusion method for detecting antagonism was used against the four common bacteria listed in **Table 1** (Nie et al., 2012). The common bacteria and endophytes were each pre-cultured overnight, and 5 mL^-1^ of each culture was centrifuged at 604 × *g* for 5 min. The pellets were resuspended in sterile phosphate buffered saline (PBS) in a laminar air flow cabinet and density adjusted to 10^8^ colony forming units (CFU) mL^-1^ by using Densicheck plus (BioMérieux, United States). A total of 200 μL of the common bacteria cell concentrate was inoculated and evenly spread by sterile cotton swaps onto the surface of the medium, and then four 5-mm-diameter pieces of sterile filter paper were placed on each corner of the petri dish. A total of 10 μL of each endophyte strain was then added dropwise to the filter paper. All plates were wrapped with parafilm, incubated at 37 ± 2°C for 24 h and observed for the inhibition of the common bacteria (Cho et al., 2007). Antibacterial activity was assessed by measuring the diameter of the clear zone of growth inhibition. An equivalent volume of sterile phosphate buffered saline (PBS) instead of the endophytic bacteria was used as a negative control.

**Table 1:** Common bacteria used in this study.

Scientific Name	Strain	Gram reaction	Source
*Staphylococcus aureus* (SA)	10786	+	China Center of Industrial Culture Collection (CICC)
*Bacillus cereus* (BC)	10451	+	China Center of Industrial Culture Collection (CICC)
*Salmonella enteritidis* (SE)	10982	-	China Center of Industrial Culture Collection (CICC)
*Escherichia coli* (EC)	GUM1.705	-	Microbial Culture Collection Center of Guangdong (GIMCC)


### Antagonistic Assays of Antifungal Activities *in Vitro*

The antifungal activity of each bacterial endophyte was screened for antagonistism against the pathogenic fungi in **Table 2** by the plate diffusion method (Sun et al., 2017). Bacterial strains were grown in ISP_2_ medium at 28°C overnight. The fungal pathogens were grown on potato dextrose agar (PDA) plates. A 5-mm agar plug containing 6-day-old mycelial growth was placed at the center of a 9-cm modified culture PDA plate, which favors for the growth of both endophytes and the pathogen. A concentrate of each endophyte was placed onto the agar surface at 8 equidistant points, 2.5 cm from the plate periphery (Loqman et al., 2009). All plates were wrapped with parafilm and incubated at 28 ± 2°C for 3–5 days and observed for the inhibition of the pathogen. Plates with pathogenic fungi alone served as a control. The percentage of growth inhibition was calculated by measuring the diameter of the inhibition zone by using the following formula:

$$\text{Inhibition rate }(\%)\;=\frac{F_{cd}-T_{cd}}{F_{cd}-F_{0}}\times 100$$

**Table 2:** Fungal pathogens used in this study.

Strain	Scientific name	Host Plant and disease	Source
F1	*Fusarium oxysporum* f. sp.	Tomato, *Fusarium* wilt	Xinjiang
F2	*Fulvia fulva* (Cooke) Cif.	Tomato, Leaf mildew	Xinjiang
F3	*Alternaria solani* Sorauer	Tomato, Early Blight	Xinjiang
F4	*Fusarium oxysporum* f. sp. Vesinfectum	Cotton, *Fusarium* wilt	Xinjiang
F5	*Verticillium dahliae* Kleb.	Cotton, *Verticillium* wilt	Xinjiang
F6	*Ceratocystis fimbriata*	Pomegranate, Pomegranate wilt	Yunnan
F7	*Colletotrichum gloeosporioides*	Cymbidium sinense, Anthracnose	Yunnan
F8	*Pestalotiopsis microspora*	Tsao-ko, pseudostem black spot disease	Yunnan
F9	*Fusarium graminearum*	Tsao-ko, Leaf spot disease	Yunnan


where F_cd_ is the fungal colony diameter on the control PDA base plate, T_cd_ is the fungal colony diameter on the experimental PDA base plate, and F_0_ is the diameter of the test fungus agar disks (5 mm) (Aeron et al., 2011). Each experiment was performed with three replicates, and the analysis was repeated to ensure consistency of the results.

### Screening for Natural Product Biosynthetic Gene Clusters by PCR Method

A total of 16 strains were used for screening for natural product biosynthetic gene clusters by PCR. Three sets of degenerate primers targeting biosynthetic genes were used for PCR amplification: KSF (5′-GTSCCSGTSSCRTGSSHYTCSA-3′) and KSR (5′-CGCTCCATGGAYCCSCARCA-3′), targeting polyketide synthase (*PKS*)*-I* KS and methyl malonyl transferase domains (Kun et al., 2010); KSαF (5′-TSGCSTGCTTGGAYGCSATC-3′) and KSαR (5′-TGGAANCCGCCGAABCCGCT-3′), targeting *PKS-II* KSα genes (Metsä-Ketelä et al., 1999); and A3F (5′-GCSTACSYSATSTACACSTCSGG-3′) and A7R (5′-SASGTCVCCSGTSCGGTAS-3′), targeting non-ribosomal peptide synthetase (*NRPS*) genes. The reactions were performed in a (BIO-RAD C1000 Thermal Cycler) in a total volume 25 μl consisting of 50 ng of genomic DNA, 10 pmol of each primer, 2.5 mM of each deoxynucleotide triphosphates, 1X PCR buffer, and 1 U of Taq DNA polymerase. Gradient PCR was performed under the following conditions: initial denaturation step at 95°C for 5 min, followed by 32 cycles of denaturation at 96°C for 1 min, annealing at 56, 62.1, and 52.5°C for *PKS-I*, *PKS-II*, and *NRPS* genes, respectively, for 1 min, and extension at 72°C for 2 min, with a final extension step at 72°C for 10 min. The amplified PCR products were analyzed by electrophoresis on 1% agarose gels with TAE buffer. A negative control without DNA template was included with each PCR.

### Digestive Enzymes

A total of 16 isolates were used for screening their ability to produce digestive enzymes. Cellulase activity was assayed with modified DSMZ^1^ medium 65 without CaCO_3_ and supplemented with carboxymethyl cellulose (5 g L^-1^; Sigma-Aldrich) in place of glucose. After incubation for 3–4 days at 28°C, plates were stained with a Congo red solution and destained with a NaCl solution (Li et al., 2018). A clear or lightly colored halo around the colonies indicated a positive reaction. Protease activity was assayed with YEM agar medium containing 5% (v/v) skim milk. After incubation for 3–4 days at 28°C, a clear halo around the bacterial colonies due to hydrolysis of milk indicated a positive reaction. Lipase enzyme activity was assayed with modified Sierra lipolysis agar supplemented with beef extract (3 g L^-1^) and ferrous citrate (0.2 g L^-1^). After autoclaving, 50 mL of Victoria Blue B solution (0.1 g 150 mL^-1^) and 10 mL of Tween-80 was added to the medium. After 5–6 days incubation at 28°C, white calcium precipitates around the bacterial colonies indicated a positive reaction (Li et al., 2018).

Colloidal chitin was prepared from commercial chitin by the method of Agrawal and Kotasthane, 2012. Chitin was hydrolyzed in concentrated HCl by stirring at 4°C overnight, followed by extraction of colloidal chitin in 200 mL of ice-cold 99% ethanol, neutralization at room temperature overnight, and centrifugation at 1677 × *g* for 10 min at 4°C. The pellet was washed with sterile distilled water by centrifugation at 2415 × *g* for 5 min at 4°C till the smell of alcohol was completely removed and the pH was 7. The colloidal chitin had a soft pasty consistency with 90% moisture and was stored at 4°C until further use. Chitinase detection medium composed of (L^-1^) 0.3 g of MgSO_4_⋅7H_2_O, 3.0 g of (NH_4_)_2_SO_4_, 2.0 g of KH_2_PO_4_, 1.0 g of citric acid monohydrate, 15 g of agar, 200 μL of Tween-80, 4.5 g of colloidal chitin, and 0.15 g of bromocresol purple and then autoclaved at 121°C for 15 min. To test for chitinase activity, inoculated plates were incubated at 25 ± 2°C and were observed for formation of a colored zone. For all the tests mentioned above, sterile nutrient agar was used as a control for bacterial growth. All experiments were performed twice with three replicates for each isolate.

### Evaluation of Biocontrol Efficacy in Pot Experiments Under Greenhouse Conditions

Five strains, XEGI33, XEGI38, XEGI44, XEGI50, and XEGI78, were selected for greenhouse experiments based on antagonistic activity against *V. dahliae in vitro* and presence of at least one biosynthetic gene and at least one digestive enzyme. *Arabidopsis thaliana* was used as a model plant and *V. dahliae* was used as a model pathogen (Maldonado-González et al., 2015). *A. thaliana* seeds with uniform shape and size were surface-sterilized with 99% ethanol for 0.5 min, and then washed with sterile distilled water 5–6 times. The seeds were placed on MS plates at 25 ± 2°C for 3–5 days. *Arabidopsis thaliana* seedlings with true stage leaves were then transplanted singly into pots of 8 cm diameter containing 60 g of sterilized soil. After 2 days of transplantation, plants were divided into five groups and each group was given different treatments. *V. dahliae* was cultivated in advance in Czapek-Dox broth at 28°C for 4 days. After 72 h of transplantation, 10 mL of the fungal mycelia suspension (10^8^ CFU mL^-1^) was spread on the soil surface above fine roots by using a sterile syringe, and then after another 48 h, 10 mL of an endophytic bacteria suspension (10^8^ CFU ml^-1^) was added, as generally described by Chen et al., 2014. Two controls were used in this experiment: seedlings treated with sterile water (CK^+^), and seedlings inoculated with the pathogen alone (CK^-^). The pots were placed in a growth chamber with the following conditions: 25–30°C, 60% humidity, and 16 h of daylight alternating with 8 h of darkness (Jiang et al., 2015). Three replicates were done for each treatment. Plant phenotypes were observed after 10 days of pathogen inoculation, while the development of wilt disease signs was recorded after 40 days.

### Disease Assessment

A disease index, based on yellowing and chlorosis of cotyledons and leaves after 40 days, was used to classify disease signs for each leaf into six grades (i.e., grade 0, 1, 2, 3, 4, and 5) (Zhang et al., 2012), and then the percentage of affected leaves was calculated and categorized (≤ 25, 25–35, 35–45, 45–55, and 55–80%). The final disease index (DI) was calculated according to the following formula: DI = [(Σ disease grades × number of infected)/(total checked plants × 5)] × 100 (Zhang et al., 2012). The DI represents a comprehensive and objective measure of plant health, with high DI values corresponding to serious infections.

### Isolation and Purification of Antimicrobial Agents

Strain XEGI50 was inoculated into 500 mL^-1^ of ISP_2_ broth at 28°C for 10 days with agitation at 120 rpm and used as a control (1). *V. dahliae* was cultivated in 500 mL^-1^ in Czapek broth at 28°C for 10 days with agitation at 120 rpm and used as a control (2). The antibiosis experiment was carried out by co-cultivation of strain XEGI50 with *V. dahliae* in 500 mL^-1^ of broth medium at 28°C for 12 days with agitation at 120 rpm. All cells were collected by centrifugation at 5000 × *g* for 10 min. The cell-free supernatant was divided into equal volumes. After that, the supernatant was adjusted to pH 7 and pH 3 with 500 mL^-1^ of 1 N HCl and an equal volume (1:1) of ethyl acetate was added and mixed by vigorous shaking for 30 min and allowed to settle. The organic solvent phase was collected and evaporated at 40°C under vacuum, using a rotary evaporator model (IKA, HB10 basic). The ethyl acetate extract was dissolved in 5 mL of Tris–Cl buffer (0.02 M, pH 7.0) and used for gas-chromatography/mass-spectrometry (GC-MS).

### Identification and Antibacterial Evaluation of Bioactive Compounds

The GC-MS analysis of the cell-free extracts was performed using a gas chromatograph (Model 7890A, Agilent, Palo Alto, CA, United States) equipped with a split-splitless injector, an Agilent model 7693 autosampler, and an Agilent HP-5MS fused silica column (5% phenyl-methylpolysiloxane, 30 m length, 0.25 mm I.D., film thickness 0.25 mm). Injecting volume was 1 μL, and the GC conditions included programmed heating from 50 to 300°C at 10°C/min, followed by 10 min at 300°C. The injector was maintained at 280°C. Helium was the carrier gas, at 1.0 mL min^-1^, and the split mode was 5:1. The GC was fitted with a quadrupole mass spectrometer with an Agilent model 5975 detector. The MS conditions were as follows: ionization energy, 70 eV; electronic impact ion source temperature, 230°C; quadrupole temperature, 150°C; scan rate, 3.2 scan/s; mass range, 50–1000 μ. The compounds were identified based on the match with their mass spectra and retention indices with the NIST/Wiley 275 library (Wiley, New York, NY, United States). Relative abundance of each feature was calculated from Total Ion Chromatogram (TIC) computationally.

### Intelligent Live Digital Imaging of Strain XEGI50 and *V. dahliae*

The morphological response of XEGI50 to *V. dahliae* was observed under a laser microscope (Olympus SZX2-ILLT, Japan) at different magnifications. Bacteria were incubated in ISP_2_ medium. *V. dahliae* was grown on PDA medium. A 6-day-old mycelial disk (5 mm) was placed at the center of a 7 cm modified culture PDA plate. The bacteria were placed at four corners on the bacterial lawn at four equidistant points of 2.5 cm from the plate periphery. All plates were wrapped with parafilm, incubated at 28 ± 2°C for 6 days, and observed for the inhibition of the pathogen. Plates with pathogenic fungi alone served as control.

## Results

### Antibacterial Activity

**FIGURE 1:**
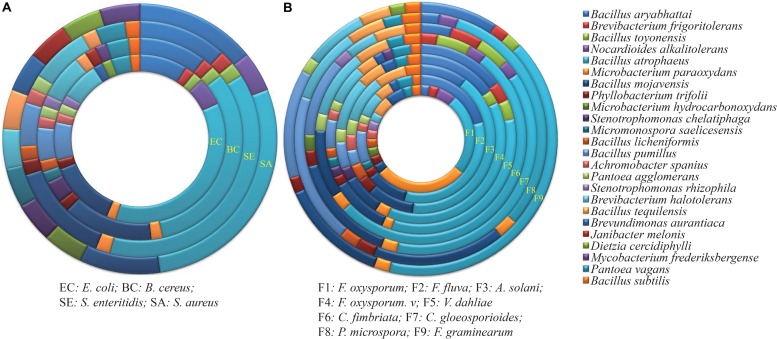
Antimicrobial activity of endophytes against **(A)** four common bacteria and **(B)** nine fungal pathogens. Each ring represents the total number of strains with activity against the pathogen, and is divided into colors based on the proportion of each genus to that total. Details on the common bacteria and fungal pathogens are shown in **Tables 1**, **2**.

A total of 114 endophytes isolated from wild populations of *G. uralensis*, representing 18 genera and 34 species, were screened for their ability to inhibit four common bacteria, representing the Gram-positive phylum *Firmicutes* and the Gram-negative phylum *Proteobacteria* (**Table 1**). Of the 114 isolates examined, 56 (49.1%) displayed inhibitory activity, ranging from 8.6 to 11.8 mm against *S. aureus*, *B. cereus*, and *S. enteritidis*; in contrast, only 14 (12.3%) were antagonistic to *E. coli*, ranging from 7.5 to 10.3 mm (**Supplementary Figures S1A,B**). These 14 strains belonged to 6 different genera, namely *Bacillus*, *Microbacterium*, *Brevibacterium*, *Phyllobacterium*, *Pantoea*, and *Stenotrophomonas*. The genus *Bacillus* showed the highest antimicrobial activity against the selected Gram-positive and Gram-negative bacteria (**Figure 1A**).

### Antifungal Activity

The inhibitory effect of all endophytic isolates was tested against the nine fungal phytopathogens listed in **Table 2**. The endophytes varied in their ability to inhibit the growth of the fungi, with the percentage of inhibition ranging from 12.3 to 75.3% (**Supplementary Figures S1C,D**). A total of 44, 75, and 34 strains (38.6, 65.8, and 29.8%) were antagonistic to the tomato pathogens *F. oxysporum* f. sp. (F1), *F. fulva* (F2), and *A. solani* (F3), respectively. For *F. oxysporum* f. sp., the largest inhibition zone (72.1%) was observed for strain XEGI74, belonging to *Bacillus halotolerans.* For *F. fulva*, the largest inhibition zone (72.0%), was observed for *Bacillus atrophaeus* strain XEGI51. And for *A. solani*, the largest inhibition zone (63.0%) was observed for strain XEGI15, belonging to *Brevibacterium frigoritolerans*.

For the pathogens causing cotton wilt diseases, the antibiosis assay demonstrated that 48 and 83 endophytic strains (42.1 and 72.8%) were antagonistic to *F. oxysporum* f. sp. Vesinfectum (F4) and *V. dahliae* (F5), respectively. For *F. oxysporum* f. sp. Vesinfectum, the largest inhibition zone (70.1%) was observed for strain XEGI9, belonging to *Nocardioides alkalitolerans*; for *V. dahliae*, the largest inhibition zone (75.5%) was observed for strain XEGI50, belonging to *B. atrophaeus*.

For the pathogens *Ceratocystis fimbriata*, *Colletotrichum gloeosporioides*, *Fusarium graminearum*, and the receently described pathogen *Pestalotiopsis microspora* (Guo J. W. et al., 2016), the antibiosis assay demonstrated that 60, 45, 57, and 40 strains (52.6, 39.5, 50.0, and 35.1%) were antagonistic to *C. fimbriata* (F6), *C. gloeosporioides* (F7), *P. microspore* (F8), and *F. graminearum* (F9), respectively. For *C. fimbriata*, the largest inhibition zone (55.4%) was observed for strain XEGI46, which belonged to *Bacillus mojavensis*. For *C. gloeosporioides*, the largest inhibition zone (48.0%) was observed for *B. atrophaeus* strain XEGI10. For *P. microspore*, the largest inhibition zone (47.9%) was observed for strain XEGI44, belonging to *Bacillus mojavensis*; and for *F. graminearum*, the largest inhibition zone (34.0%) was observed for strain XEGI39, which belonged to *B. atrophaeus*. Based on our investigation, members of the genus *Bacillus* had the highest antagonistic activity of any of the bacterial genera (**Figure 1B**), and *V. dahliae* was the most susceptible fungal pathogen.

### Analysis of Putative Biosynthetic Genes

The presence of putative biosynthetic genes encoding *PKSs* and *NRPS* of peptide antibiotics were investigated in the 16 strains exhibiting the strongest antimicrobial activities by using 3 sets of degenerate PCR primers. Among the 16 strains, 12 were positive for *PKSs* genes (**Table 3**); the translated amino acid sequences of these *PKS-I* genes shared moderate to high amino acid identity (54–99%) with those from members of the phylum *Actinobacteria*. A total of seven were positive for *NRPS* genes (**Table 3**). The *NRPS* sequences shared 40–70% amino acid sequence identities with peptide synthetase genes of the genus *Bacillus*, except the sequence from *Microbacterium paraoxydans* strain XEGI12, which was most closely related to enzymes of genus *Microbacterium*. Moreover, of the 16 isolates, 7 strains were positive for amplification of all 3 biosynthetic genes; 2 strains were positive for 2 genes; 4 strains were positive for only 1 gene; and only 3 strains were negative for all 3 *PKS* and *NRPS* genes (**Table 3** and **Supplementary Figure S2**).

**Table 3:** Presence of biosynthetic genes (*PKSI*, *PKSII*, and *NRPS*) and digestive enzyme activity of the 16 most active strains.

Strain	Accession Number	Closest species in 16S rRNA gene sequences database	*PKSI*	*PKS II*	*NRPS*	Protease^a^	Cellulase^b^	Lipase^c^	Chitinase
XEGI10	KY127316	*Bacillus atrophaeus*	+	+	+	+++	-	+	+
XEGI12	KY127318	*Microbacterium paraoxydans*	+	+	+	-	-	-	-
XEGI14	KY127320	*Bacillus atrophaeus*	+	+	+	++	-	++	-
XEGI15	KY127321	*Brevibacterium frigoritolerans*	-	-	-	-	-	++	-
XEGI16	KY127322	*Bacillus mojavensis*	+	-	-	++	+	+++	-
XEGI33	KY127337	*Bacillus atrophaeus*	+	+	+	++	-	-	+
XEGI35	KY127339	*Bacillus atrophaeus*	+	+	-	++	-	-	-
XEGI38	KY127341	*Bacillus atrophaeus*	+	+	-	+++	-	-	-
XEGI44	KY127347	*Bacillus mojavensis*	-	+	-	+++	-	+	-
XEGI45	KY127348	*Bacillus atrophaeus*	+	-	-	+	+	+	-
XEGI46	KY127349	*Bacillus mojavensis*	+	+	+	+	-	+	-
XEGI50	KY127353	*Bacillus atrophaeus*	+	+	+	+++	-	++	+
XEGI56	KY127359	*Bacillus mojavensis*	+	-	-	++	+	+	+
XEGI74	KY127370	*Bacillus halotolerans*	-	-	-	+	++	++	-
XEGI78	KY127374	*Bacillus atrophaeus*	+	+	+	++	-	+	-
XEGI95	KY127390	*Bacillus tequilensis*	-	-	-	+	+	+	-


*^*a*^Protease production: “-” no production; “+” weak halo around the colony (0.50–1.50 cm); “++” clear halo around the colony (1.50–2.00 cm); “+++” strong halo around the colony (2.00–3.00 cm). ^*b*^Cellulase production: “-” no production; “+” weak halo around the colony (1.00–1.50 cm); “++” clear halo around the colony (1.50–2.51 cm). ^*c*^Lipase production: “-” no production; “+” weak halo around the colony (1.00–1.50 cm); “++” clear halo around the colony (1.50–2.00 cm); “+++” strong halo around the colony (2.00–2.50 cm).*

### Digestive Enzyme Activity

The same 16 strains were assayed for protease, cellulase, lipase, and chitinase, which are potentially involved in lysis of phytopathogens or modulation of virulence factors. Some of the tested strains produced one or more lytic enzyme, as assessed by a change in pH in plates containing soluble chitin and bromocresol purple (chitinase) or by the diameter of the halo zone on media containing skim milk (protease), carboxymethyl cellulose (cellulase), or Sierra lipolysis agar (lipase) (**Table 3** and **Supplementary Figure S3**). Proteases and lipases were the most common enzymes detected. The *Bacillus* strains were more active in these assays than the other two genera tested.

### Biological Control of *V. dahliae* in Growth Chambers

**FIGURE 2:**
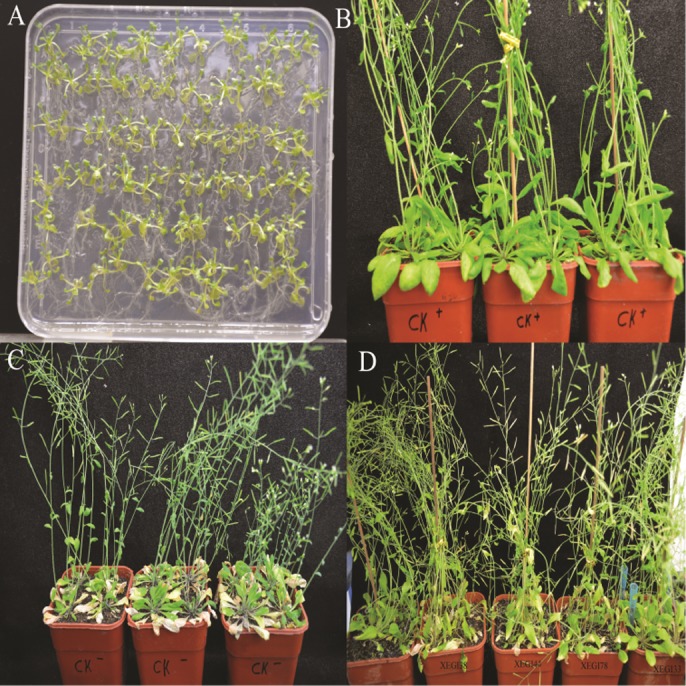
Defense response to *V. dahliae* in *Arabidopsis thaliana* plants **(A)** *A. thaliana* seedling; **(B)** *A. thaliana* without inoculation of *V. dahliae*; **(C)** Response of *A. thaliana* to *V. dahliae*; **(D)** Response of *A. thaliana to V. dahliae* inoculated with different endophytic strains after 5 weeks.

**FIGURE 3:**
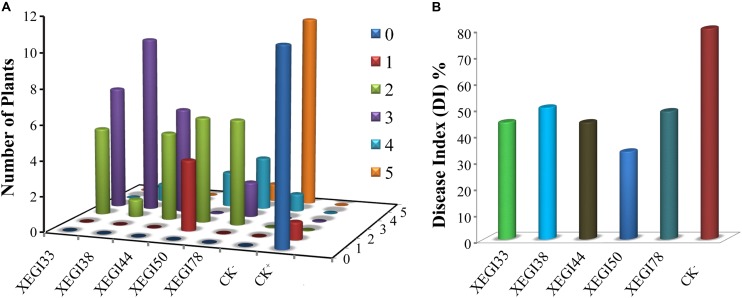
Effect of antagonistic isolates on disease grades and disease index of *Arabidopsis thaliana* plants to *V. dahliae* over 5 weeks after inoculation of the pathogen. **(A)** Signs were rated along a scale that assigned disease grades ranging from ‘0’ to ‘5’ (0: no signs, 1: ≤ 25, 2: 25–35, 3: 35–45, 4: 45–55, 5: 55–80%); **(B)** disease index of *Arabidopsis thaliana*.

An infection time course of *A. thaliana* and *V. dahliae* was developed by evaluating plant disease signs over a 5-week period. The first signs developed within 7 days of inoculation, including yellowing of leaves, and leaf chlorosis, which began with older leaves and progressed to younger leaves. Severe signs were seen on the leaves of the plants challenged with *V. dahliae* in the absence of endophytes (**Figure 2**). After 5 weeks, the control plantlets under pathogen-challenged conditions showed severe signs, while those inoculated with endophytes showed mild signs of disease. Although all tested endophytes conferred some degree of *Verticillium* wilt resistance, the distribution of disease grades varied dramatically (**Figure 3A**). The disease severity index (DSI) in plantlets treated with strains XEGI33, XEGI38, XEGI44, XEGI50, and XEGI78 were each significantly reduced (44.5, 50.0, 44.5, 33.3, and 48.6%), compared to those grown in the absence of the biocontrol agent, which was 78.0% (**Figure 3B**). XEGI50 conferred the best protection (33.3% DSI) by suppressing yellowing other and wilt signs. This result suggested that XEGI50 could slow disease development, and the expression of signs were delayed compared with other treatments.

### Detection of Bioactive Compounds by GC-MS Analysis

To determine the prevalent organic compounds produced by the most bioactive strain, *B. atrophaeus* XEGI50, ethyl acetate extracts of cell supernatant buffered at pH 7 and pH 3 were concentrated from cultures of strain XEGI50, *V. dahlia*, and a co-culture. GC–MS analysis showed that different features (putative compounds) were produced at pH 7 and pH 3. Features were tentatively identified based on comparison of spectra avaiable through the National Institute of Standards and Technology (NIST) database, and biological activities were interpreted primarily based on Dr. Duke’s Phytochemical and Ethnobotanical Databases created by Dr. Jim Duke of the Agricultural Research Service/USDA.

**FIGURE 4:**
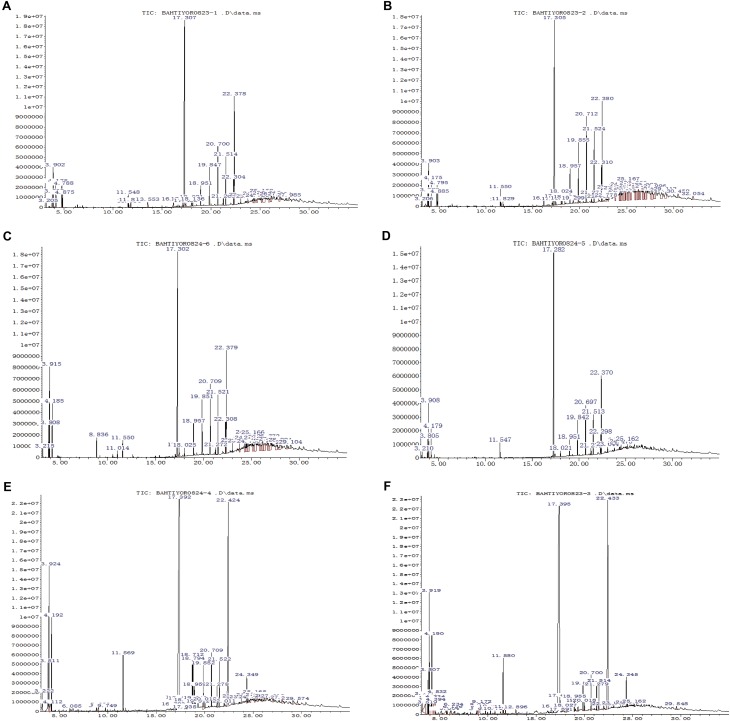
GC-MS analysis of bioactive compound of ethyl acetate extract sample. **(A)** The crude extract of XEGI50 at pH 7; **(B)** The crude extract of XEGI50 at pH 3; **(C)** The crude extract of *V. dahliae* at pH 7; **(D)** The crude extract of *V. dahliae* at pH 3; **(E)** Antibiosis crude extract of XEGI50 and *V. dahliae* mixture at pH 7; **(F)** Antibiosis crude extract of XEGI50 and *V. dahliae* mixture at pH 3.

The GC-MS analysis of crude extracts buffered at pH 7 from strain XEGI50 cultivated alone revealed at least 21 features (**Supplementary Table S1**) and 36 features at pH 7 (**Supplementary Table S2**). Nine distinctive features in the pH 7 extract, compared to pH 3, were present at RT 3.897, 4.171, 17.302, 19.849, 20.701, 21.511, 22.301, 22.374, and 24.331, suggesting the presence of benzene, 1,3-dimethyl-; o-xylene; dibutyl phthalate; heptadecane; eicosane; tetracosane; pentacosane; bis (2-ethylhexyl) phthalate; and decanedioic acid, bis (2-ethylhexyl) ester (**Figure 4A**). The pH 3 extract showed three major peaks that were different from the pH 7 extract at RT 3.908, 18.954, and 19.859, suggestive of p-xylene; heneicosane; and docosane (**Figure 4B**).

The GC-MS analysis of crude extracts from *V. dahliae* cultivated alone showed 32 compounds in the pH 7 extract (**Supplementary Table S3**) and 17 compounds in the pH 3 extract (**Supplementary Table S4**). Six major features were obtained from the pH 7 extract at RT 3.802, 3.918, 4.181, 17.302, 20.711, and 22.384, suggestive of ethylbenzene; p-xylene; benzene 1,3-dimethyl-; dibutyl phthalate; heptadecane; and bis (2-ethylhexyl) phthalate (**Figure 4C**). The pH 3-buffered extract from *V. dahliae* showed one major peak are different from the crude extract at pH 7 at RT 22.374, suggestive of phthalic acid, di (2-propylpentyl) ester (**Figure 4D**).

The GC-MS resolved several features in the extracts of XEGI50 and *V. dahliae* mixture: 37 compounds from the pH 7 extract (**Supplementary Table S5**) and 32 compounds from pH 3 extract (**Supplementary Table S6**). A total of 12 major peaks were obtained from the pH 7 extract at RT 3.813, 3.929, 4.192, 11.568, 17.397, 18.712, 18.796, 19.848, 20.711, 21.521, 22.426, and 24.352, suggestive of ethylbenzene; p-xylene; dimethyl phthalate; 1,2-benzenedicarboxylic acid; bis (2-methyl propyl) ester; 9,12-octadecadienoic acid (Z,Z)- methyl ester; 9-octadecenoic acidmethyl ester, (E)-; eicosane; heptadecane; tetracosane; bis (2-ethylhexyl) phthalate; and decanedioic acid, bis (2-ethylhexyl) ester (**Figure 4E**). The pH 3 extract from the co-culture contained most of the same compounds, and one additional feature at RT 4.192, suggestive of o-xylene (**Figure 4F**).

Many compounds tentatively identified in the GC-MS analysis are known to have antimicrobial activity. Therefore, to determine the prevalent compounds that were induced by co-cultivation, putative compounds were compared between the three different datasets. At least 13 compounds were detected only in the co-culture, consisting of mainly fatty acid esters, phenols, alkanes, alkenes, and aromatic chemicals. Four of them showed high peaks at RT 17.397, 18.712, 18.796, and 24.35, suggestive of 1,2-benzenedicarboxylic acid butyl 2-ethylhexyl ester; 9,12-octadecadienoic acid (Z,Z)-, methyl ester; 9-octadecenoic acid, methyl ester, (E)-; and decanedioic acid, bis (2-ethylhexyl) ester (**Figures 4E,F**). In addition, several minor peaks were present, including butanoic acid, 2-hydroxy-3-methyl-; eicosane9-cyclohexyl-, heptadecanoic acid, 16-methyl-, methyl ester; hexadecanoic acid, methyl ester; hexanedioic acid, dioctyl ester; naphthalene, 1-methyl-; naphthalene, 2-methyl-; *n*-decanoic acid; pentanoic acid, 4-methyl-; and picolinamide (**Supplementary Tables S5**, **S6**).

### Defense Response of Strain XEGI50 to *V. dahlia* via Laser Microscopy

**FIGURE 5:**
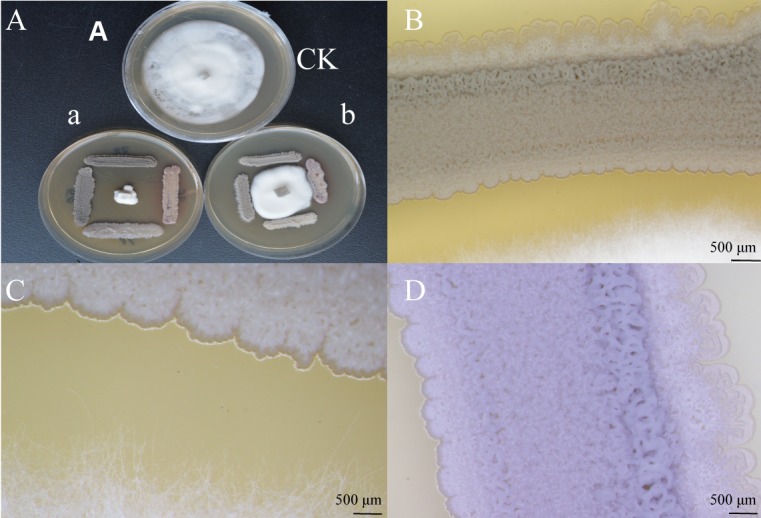
Intelligent live digital imaging of the response of endophytic strain XEGI50 to *V. dahliae* under a laser microscope. **(A)** *In vitro* evaluation of antagonistic activity of strain XEGI50; **(a)** *V. dahliae* could not grow after seven days of incubation with antagonistic strains, **(b)** *V. dahliae* grows after three days then we inoculate the antagonistic stains at four sides of the agar plate. **(B)** Response of strain XEGI50 to *V. dahliae* at 1.25× magnification; **(C)** Response of strain XEGI50 to *V. dahliae* at 4× magnification showed white powder appeared only on one the side which is facing the pathogenic fungi; **(D)** Different changes of the behavior of strain XEGI50 on the both sides due to the stress at 2.5× magnification.

The results showed that *V. dahliae* could not grow after 7 days of incubation with antagonistic strains (**Figures 5Aa,b**). The morphological response of XEGI50 to *V. dahliae* was observed under a laser microscope at different magnifications (1.25×, 2.5×, 4×). The microscopic characteristics of strains XEGI50 based on laser microscopy showed that at 1.25× magnification the endophytic strain XEGI50 was able to control the growth of the fungal mycelium after 5 days of cultivation (**Figure 5B**) and at 4×, a white powder appeared only on the side facing the pathogenic fungi, we hypothesize that strain XEGI50 may secrete some antifungal compound (**Figure 5C**). In addition, at the same magnification of 2.5×, strain XEGI50 looked intense at the bacteria/fungi interface, but expanded in the direction away from *V. dahliae* (**Figure 5D**).

## Discussion

Biological control of plant pathogens using microorganisms can be a safe, cost-effective, and efficient method for suppressing plant diseases. Endophytes accociated with medicinal plants are rich sources of secondary metabolites with antimicrobial activity, and they spend their whole life cycle within plant tissues without causing any infections or signs of disease (Bacon and White, 2000; Saikkonen et al., 2004). In addition, it has also been documented that endophytes associated with medicinal plants may produce the same metabolites *in vitro* and within host plant tissue (Kusari et al., 2013; Dos Santos et al., 2016). In the present study, we analyzed the antimicrobial activity of a diverse collection of endophytes previously isolated from wild populations of *G. uralensis* (Li et al., 2018), consisting of 18 genera and 34 species, with a goal of determining which strains offer the strongest antagonistic activities against pathogens, and to identify microbial products that may confer these antagonistic activities.

*In vitro* screens for antagonistic activity were conducted by co-cultivating the *G. uralensis* endophytes with common fungal pathogens of tomato (*F. oxysporum* f. sp., *F. fulva*, *A. solani*), cotton (*F. oxysporum* f. sp. Vesinfectum, *V. dahliae*), pomegranite (*C. fimbriata*), *Cymbidinium* (*C. gloeosporioides*), and Tsao-ko (*P. microspora* and *F. graminearum*), and the common bacteria *S. aureus*, *B. cereus*, *S. enteritidis*, and *E. coli*. In these assays, a significant fraction of the endophytic bacteria displayed antagonistic effects. The genus *Bacillus* (**Figures 1A,B**) was the dominant genus, with high antimicrobial activity against all indicator pathogens used in this study. Several studies have observed similar trends. For example, other studies have demonstrated that *Bacillus* strains associated with medicinal plants exhibit antibacterial activity against common bacteria such as *S. aureus*, *Streptococcus pyogenes*, *Pseudomonas aeruginosa*, and *E. coli* (Slepecky and He, 1992; Nejatzadeh-Barandozi, 2013; Egamberdieva et al., 2017a). Moreover, other studies have shown that *Bacillus* strains isolated from medicinal plants inhibited the mycelial growth of diverse vascular wilts caused by pathogenic fungi (Cho et al., 2002; Ebrahimi et al., 2010; Jiang et al., 2015; Akinsanya et al., 2015; Gao et al., 2017).

In our study, 16 strains showing antagonistic activity toward at least 7 pathogenic fungi and at least 1 common bacterium, were screened for the presence of *PKS-I*, *PKS-II*, and *NRPS* gene clusters to determine whether their genomes contain these genes. These 16 strains consisted of 1 strain each of *Microbacterium paraoxydans* and *B. frigoritolerans*, and 14 strains of *Bacillus*, belonging to *Bacillus atrophaeus* (8 strains), *Bacillus mojavensis* (four strains), *Bacillus halotolerans* (1 strain), and *Bacillus tequilensis* (1 strain). Only 12, 10, and 7 strains were positive for PCR amplification of fragments of *PKS-I*, *PKS-II*, and *NRPS* genes, respectively (**Table 3**). Several isolates had antimicrobial activity, but biosynthetic genes were not successfully amplified; they were *B. frigoritolerans* XEGI15, *Bacillus halotolerans* XEGI74, and *Bacillus tequilensis* XEGI95. The absence of amplification products may be due to the lack of natural product biosynthetic genes or because they contain divergent or novel genes that are not recognized by the degenerate primers used in this study (Courtois et al., 2003; Finking and Marahiel, 2004). Moreover, not all *NRPS* genes are involved in the biosynthesis of bioactive secondary metabolites, and there might be additional types of bioactive agents or mechanisms that may be involved in the generation of antimicrobial activities (Schneemann et al., 2010; Liu L. et al., 2016).

The 16 most antagonistic strains each produced at least 1 digestive enzyme, such as protease, cellulase, lipase, and chitinase (**Table 3**). Thus, these endophytes may protect the plant from fungi and insects by degrading the fungal cell wall or cell membrane, by degrading cell membrane proteins or extracellular virulence factors, or by stimulating systemic resistance in plants (Frankowski et al., 2001). In our previous investigation, endophytic bacteria isolated from the medicinal plants *Ferula songorica*, *Hypericum perforatum*, *and Ferula sinkiangensis* secreted similar digestive enzymes (Liu Y.-H. et al., 2016; Liu et al., 2017). Moreover, similar work done by Egamberdieva et al. (2017a) reported that endophytic bacteria associated with the medicinal plant *Ziziphora capital* were able to produce chitinolytic enzymes.

*Verticillium dahliae*, which infects cotton and several other plants, causes wilt diseases and crop losses of varying severity as well as natural ecosystems (Erdogan and Benlioglu, 2010). The signs of *Verticillium* wilt disease start with yellowing followed by chlorosis and necrosis of leaves (Ting, 2014). *Arabidopsis thaliana* is an excellent tool to identify traits involved in *V. dahliae* biocontrol by endophytic bacteria (Maldonado-González et al., 2015). In the current study, five endophytic bacterial strains, XEGI33, XEGI38, XEGI44, XEGI50, and XEGI78, representing *B. atrophaeus* and *B. mojavensis*, were selected for growth chamber experiments to suppress *V. dahliae* pathogenesis in *A. thaliana*. These five strains were chosen based on *in vitro* effects against a large number of pathogens. All five strains seemed to colonize *A. thaliana* and significantly reduced the DSI (**Figure 3**). In accordance with these results, several previous studies have shown that endophytic *Bacillus* species control fungal pathogens, including *B. mojavensis*, *B. subtilis* (Bacon and Hinton, 2002; Bacon et al., 2005; Cazorla et al., 2007), *B. tequilensis*, *B. velezensis*, *B. amyloliquefaciens* (Alvarez et al., 2012; Akinsanya et al., 2015; Gao et al., 2017), and *B. megaterium*, (Cho et al., 2002; Lin et al., 2013). In addition, Guo Y. et al. (2016) isolated and characterized *B. atrophaeus* strain OSY-7LA and showed that it exhibited a strong antagonistic activity against *Listeria innocua*, a food-borne pathogen that can survive at extreme pH, temperature, and high salt concentration.

Among the endophytes, *B. atrophaeus* XEGI50 was selected for exometabolomic studies by GC-MS, based on its ability to decrease the DSI and suppress the growth of *V. dahliae.* A total of 13 features that were expressed only in co-cultures of XEGI50 and *V. dahliae* were tentatively identified as compounds with known antimicrobial, antiphrastic, antitumor, and anticancer properties. Among these compounds, four of them were major peaks in cell-free extracts from the co-culture, suggesting they play an important role in antimicrobial activities; these compounds were 1,2-benzenedicarboxylic acid, butyl 2-ethylhexyl ester (Kavitha et al., 2010); 9,12-octadecadienoic acid (Z,Z)-, methyl ester (Sermakkani and Thangapandian, 2012); 9-octadecenoic acid, methyl ester, (E)-; and decanedioic acid, bis (2-ethylhexyl) ester (Tambekar et al., 2014). In addition, several putative antimicrobial compounds were identified as minor peaks: eicosane, 9-cyclohexyl (Hsouna et al., 2011); heptadecanoic acid, 16-methyl-, methyl ester (Kandasamy et al., 2012); hexadecanoic acid, methyl ester (Chandrasekaran et al., 2011); hexanedioic acid, dioctyl ester (Rodríguez-Meizoso et al., 2010); naphthalene, 1-methyl; naphthalene, 2-methyl- (Rokade and Sayyed, 2009); and pentanoic acid, 4-methyl- (Sharma et al., 2016). Since these compounds were not produced by pure cultures of XEGI50, they were likely induced by the presence of fungal pathogens such as *V. dahliae*, and very likely play a role in antagonism of pathogens.

The genus *Bacillus* is well known for the natural production of secondary metabolites with antibacterial and antifungal activities and has a strong potential to control plant diseases (Lodewyckx et al., 2002; Cavaglieri et al., 2005; Radhakrishnan et al., 2017). This study further illustrates its potential role as a biological agent for controlling phytopathogens. In recent years, the development of biological agents derived from *Bacillus* isolates, such as “Avogreen” (Korsten et al., 1997; Janisiewicz and Korsten, 2002) and “Shemer” (Droby, 2005), has been shown to be effective biocontrols against some plant diseases. In the present study, we provide insights about plant beneficial traits of culturable endophytic bacteria associated with the medicinal plant *G. uralensis.* Our results may provide a new biological control agent for controlling *V. dahliae* and improve our understanding of the biocontrol mechanism of natural endophytes belonging to the genus *Bacillus*. These results support the development of natural products that may minimize the need for the application of chemical fungicides, which would be an environmentally friendly approach and preserve biological resources in a sustainable agricultural system.

## Conclusion

Our study revealed that natural endophytes of natural populations of the medicinal plant *G. uralensis* have a variety of antimicrobial activities *in vitro* and *in vivo*. The genus *Bacillus*, particularly *B. atrophaeus* and *B. mojavensis*, were the most effective biocontrol agents, with most strains exhibiting broad antibacterial and antifungal activities. Most of these bacteria contained genes for *PKS* and non-ribosomal proteins, both known to encode antimicrobial compounds, as well as extracellular digestive enzymes that may destroy or neutralize a variety of pathogens, including chitinases, cellulases, lipases, and proteases. Strain XEGI50, which belongs to *Bacillus atrophaeus*, was the most effective at reducing disease signs in *A. thaliana* in plant growth chambers. XEGI50 produces at least 13 compounds when co-cultivated with *V. dahlia*, many of which were putatively identified as compounds with known antimicrobial effects. To our knowledge, this is the first report establishing that *B. atrophaeus* produces bioactive compounds with antimicrobial activity. Future studies are needed to unequivocally identify these compounds, to establish their effects individually in model plant systems, and to better understand genetic and biochemical pathways for synthesis of these compounds.

## Author Contributions

OAAM, LL and W-JL participated in the design of the study, performed all the experiments and the interpretation of results, and wrote the manuscript. J-BM and SH participated in the antimicrobial experiments *in vitro* condition and provided the pathogenic bacteria, and also conducted the greenhouse experiments and data analysis. LX and J-WG conducted to antifungal activity *in vitro* condition and also provided some of the pathogenic fungi. BR did the GC-MS analyses and data analysis. Y-HL helped for preparing enzymes activity test, microscopic analysis, and PCR works. BH revised the revision and improve the language and structure of the manuscript. W-JL and OAAM revised the manuscript and supervised the hall experiments. All authors edited and critically revised the manuscript.

## Conflict of Interest Statement

The authors declare that the research was conducted in the absence of any commercial or financial relationships that could be construed as a potential conflict of interest.

## Footnotes

1 http://www.dsmz.de/microorganisms/medium/pdf/DSMZ_Medium65.pdf

## Supplementary Material

FIGURE S1 *In vitro* evaluation of antagonistic activity of endophytic bacterial isolates associated with *G. uralensis*. **(A)** *Bacillus cereus* (BC); **(B)** *Salmonella enteritidis* (SE); **(C)** *Verticillium dahliae*; **(D)** *Fusarium oxysporum.*

FIGURE S2 Agarose gel showing PCR results for *PKS-I*, *PKS-II*, and *NRPS* from selected strains. (M: Marker 1,500 bp).

FIGURE S3 Ability of endophytes strains to produce lytic enzymes. **(A)** breakdown of chitin into N-acetyl glucosamine causes an increase in pH and a change from yellow to purple zone around bacterial colony; **(B)** control; **(C)** clear zone formation in cellulose medium around bacterial colony; **(D)** Clear zone formation in protases medium around bacterial colony.

TABLE S1 GC-MS identified components of the crude extract of XEGI50 at pH7 (Volatile compounds are listed in ascending order of Retention Time).

TABLE S2 GC-MS identified components of the crude extract of XEGI50 at pH3 (Volatile compounds are listed in ascending order of Retention Time).

TABLE S3 GC-MS identified components of the crude extract of *V. dahliae* at pH7 (Volatile compounds are listed in ascending order of Retention Time).

TABLE S4 GC-MS identified components of the crude extract of *V. dahliae*at pH3 (Volatile compounds are listed in ascending order of Retention Time).

TABLE S5 GC-MS identified components of the antibiosis crude extract of XEGI50 and *V. dahliae* mixture at pH7 (Volatile compounds are listed in ascending order of Retention Time).

TABLE S6 GC-MS identified components of the antibiosis crude extract of XEGI50 and *V. dahliae* mixture at pH3 (Volatile Compounds are listed in ascending order of Retention Time).
