# Supplementary figures and images for: Evaluation of the Antimicrobial Activity of Endophytic Bacterial Populations From Chinese Traditional Medicinal Plant Licorice and Characterization of the Bioactive Secondary Metabolites Produced by Bacillus atrophaeus Against Verticillium dahliae
Source: Front Microbiol. 2018 May 9;9:924. doi: 10.3389/fmicb.2018.00924 (PMC5954123; doi:10.3389/fmicb.2018.00924)

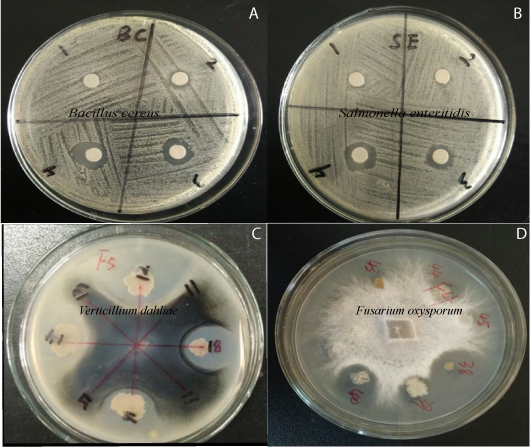

Supplement: FIGURE S1 — In vitro evaluation of antagonistic activity of endophytic bacterial isolates associated with G. uralensis. (A) Bacillus cereus (BC); (B) Salmonella enteritidis (SE); (C) Verticillium dahliae; (D) Fusarium oxysporum. [file Image_1.TIF]

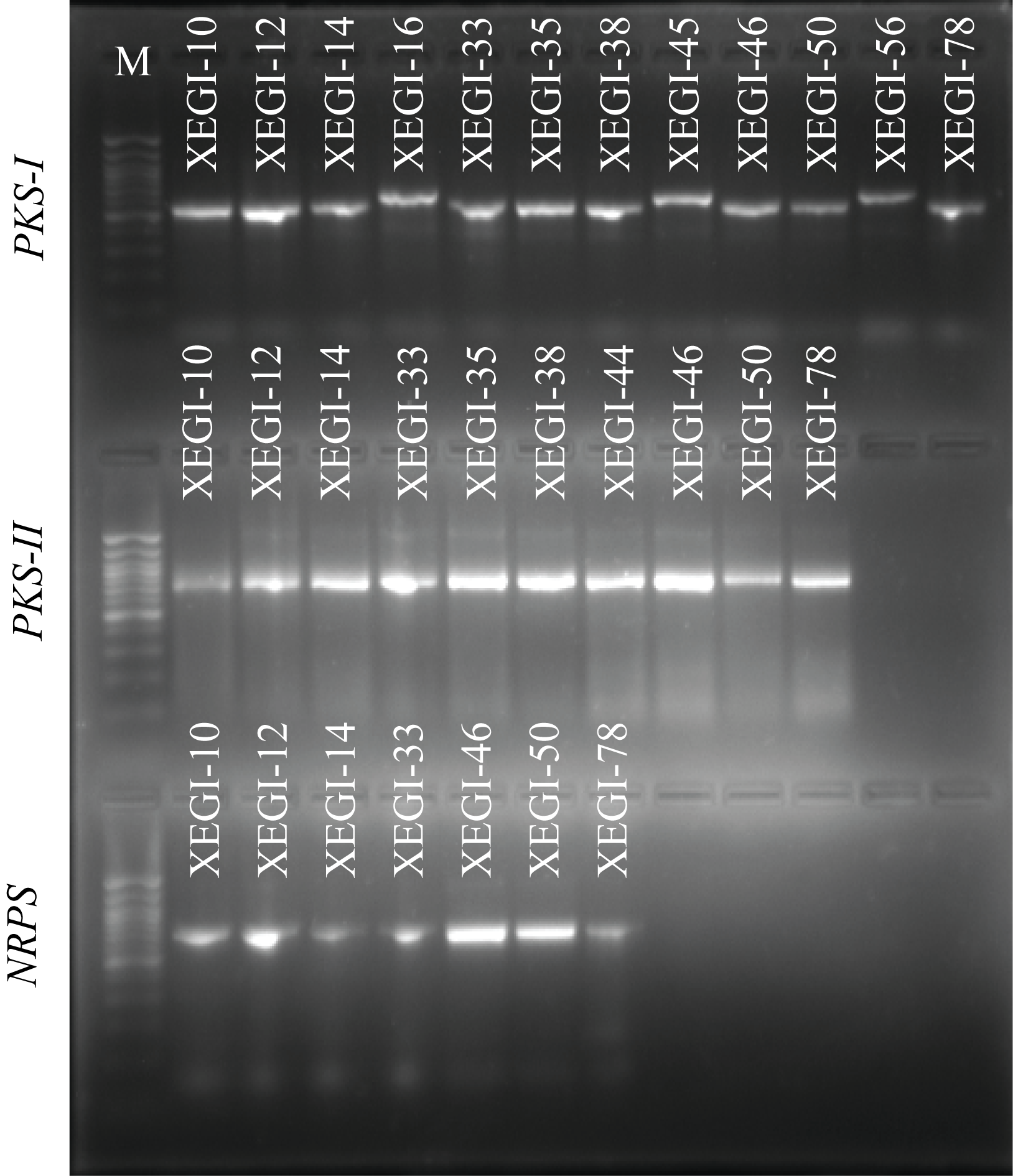

Supplement: FIGURE S2 — Agarose gel showing PCR results for PKS-I, PKS-II, and NRPS from selected strains. (M: Marker 1,500 bp). [file Image_2.TIF]

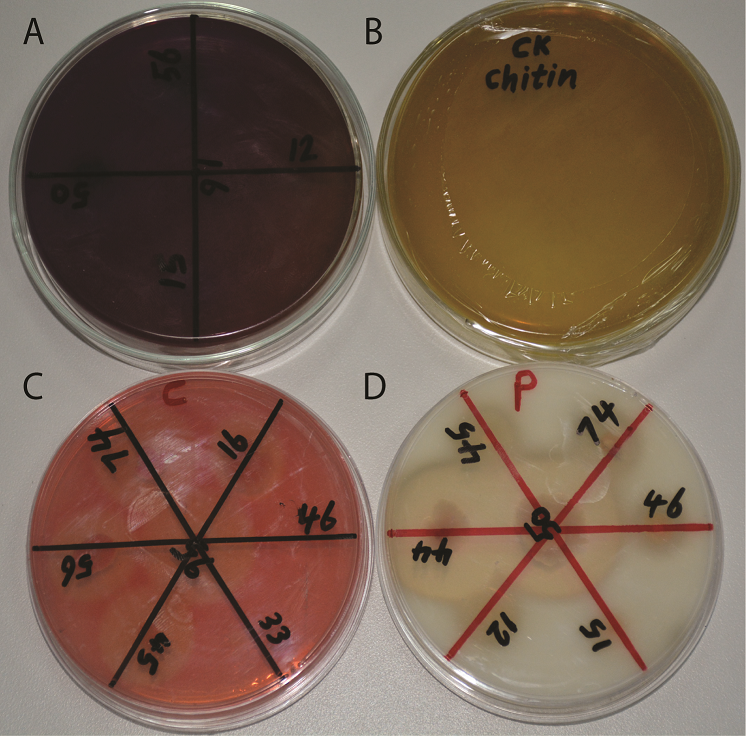

Supplement: FIGURE S3 — Ability of endophytes strains to produce lytic enzymes. (A) breakdown of chitin into N-acetyl glucosamine causes an increase in pH and a change from yellow to purple zone around bacterial colony; (B) control; (C) clear zone formation in cellulose medium around bacterial colony; (D) Clear zone formation in protases medium around bacterial colony. [file Image_3.TIF]
