# Supplementary material for: Evaluation of the Antimicrobial Activity of Endophytic Bacterial Populations From Chinese Traditional Medicinal Plant Licorice and Characterization of the Bioactive Secondary Metabolites Produced by Bacillus atrophaeus Against Verticillium dahliae
Source: Front Microbiol. 2018 May 9;9:924. doi: 10.3389/fmicb.2018.00924 (PMC5954123; doi:10.3389/fmicb.2018.00924)
Supplement: TABLE S1 — GC-MS identified components of the crude extract of XEGI50 at pH7 (Volatile compounds are listed in ascending order of Retention Time). [file Table_1.DOC]

**Table.S1:** GC-MS identified components of the crude extract of XEGI50 at pH7. (Volatile compounds are listed in ascending order of Retention Time).

| **NO** | **Retention Time (min)** | **Compounds** | **Percentage**  **Match %** | **Molecular formula** | **Molecular Weight** |
| --- | --- | --- | --- | --- | --- |
| 1 | 3.203 | Acetic acid, butyl ester | 83 | C_6_H_12_O_2_ | 116.1 |
| 2 | 3.803 | Ethylbenzene | 91 | C_8_H_10_ | 106.1 |
| 3 | 3.897 | Benzene, 1,3-dimethyl- | 97 | C_8_H_10_ | 106.1 |
| 4 | 4.171 | o-Xylene | 97 | C_8_H_10_ | 106.1 |
| 5 | 11.547 | Dimethyl phthalate | 97 | C_10_H_10_O_4_ | 194.1 |
| 6 | 17.123 | n-Hexadecanoic acid | 98 | C_16_H_32_O_2_ | 256.4 |
| 7 | 17.302 | Dibutyl phthalate | 94 | C_16_H_22_O_4_ | 278.3 |
| 8 | 17.428 | n-Hexadecanoic acid | 99 | C_16_H_32_O_2_ | 256.4 |
| 9 | 18.018 | Eicosane | 99 | C_20_H_42_ | 282.5 |
| 10 | 18.954 | Heneicosane | 98 | C_21_H_44_ | 296 |
| 11 | 19.849 | Heptadecane | 96 | C_17_H_36_ | 240.4 |
| 12 | 20.701 | Eicosane | 98 | C_20_H_42_ | 282.5 |
| 13 | 21.269 | Hexanedioic acid, bis(2-ethylhexyl) ester | 91 | C_22_H_42_O_4_ | 370.5 |
| 14 | 21.511 | Tetracosane | 99 | C_24_H_5_ | 338.6 |
| 15 | 22.3 | Pentacosane | 99 | C_25_H_52_ | 352.6 |
| 16 | 22.374 | Bis(2-ethylhexyl) phthalate | 98 | C_24_H_38_O_4_ | 390.5 |
| 17 | 23.037 | Heneicosane | 95 | C_21_H_44_ | 296.5 |
| 18 | 23.773 | Tetracosane | 97 | C_24_H_50_ | 338.6 |
| 19 | 24.468 | Octacosane | 97 | C_28_H_58_ | 394.7 |
| 20 | 26.151 | Dotriacontyl pentafluoropropionate | 93 | C_35_H_65_F_5_O_2_ | 612.8 |
| 21 | 27.003 | Tetrapentacontane, 1,54-dibromo- | 93 | Not found in the database | |

**Table.S2:** GC-MS identified components of the crude extract of XEGI50 at pH3. (Volatile compounds are listed in ascending order of Retention Time).

| **NO** | **Retention Time (min)** | **Compounds** | **Percentage**  **Match %** | **Molecular formula** | **Molecular Weight** |
| --- | --- | --- | --- | --- | --- |
| 1 | 3.203 | Acetic acid, butyl ester | 83 | C_6_H_12_O_2_ | 116.1 |
| 2 | 3.802 | Benzene, 1,3-dimethyl- | 91 | C_8_H_10_ | 106.1 |
| 3 | 3.908 | p-Xylene | 97 | C_8_H_10_ | 106.1 |
| 4 | 4.171 | Benzene, 1,3-dimethyl- | 97 | C_8_H_10_ | 106.1 |
| 5 | 11.546 | Dimethyl phthalate | 93 | C_16_H_22_O_4_ | 278.3 |
| 6 | 17.123 | n-Hexadecanoic acid | 98 | C_16_H_32_O_2_ | 256.4 |
| 7 | 17.302 | 1,2-Benzenedicarboxylic acid, bis(2-methylpropyl) ester | 94 | C_20_H_30_O_4_ | 334.4 |
| 8 | 17.439 | n-Hexadecanoic acid | 99 | C_16_H_32_O_2_ | 256.4 |
| 9 | 18.028 | Eicosane | 99 | C_20_H_42_ | 282.5 |
| 10 | 18.407 | Myo-inositol, hexaacetate | 90 | C_18_H_24_O_12_ | 432.3 |
| 11 | 18.954 | Heneicosane | 99 | C_21_H_44_ | 296.5 |
| 12 | 19.301 | Octadecanoic acid | 96 | C_18_H_36_O_2_ | 284.4 |
| 13 | 19.859 | Docosane | 98 | C_22_H_46_ | 310.6 |
| 14 | 20.711 | Eicosane | 98 | C_20_H_42_ | 282.5 |
| 15 | 21.269 | Hexanedioic acid, bis(2-ethylhexyl) ester | 93 | C_22_H_42_O_4_ | 370.5 |
| 16 | 21.521 | Tetracosane | 99 | C_24_H_50_ | 338.6 |
| 17 | 22.016 | 2-Methyltetracosane | 98 | C_25_H_52_ | 352.6 |
| 18 | 22.31 | Pentacosane | 99 | C_25_H_52_ | 352.6 |
| 19 | 22.384 | Bis(2-ethylhexyl) phthalate | 98 | C_24_H_38_O_4_ | 390.5 |
| 20 | 22.773 | 1-Bromodocosane | 87 | C_22_H_45_Br | 389.4 |
| 21 | 23.047 | Tetracosane | 97 | C_24_H_50_ | 338.6 |
| 22 | 23.773 | Tetracosane | 96 | C_24_H_50_ | 338.6 |
| 23 | 24.225 | Octadecane, 1-iodo- | 95 | C_18_H_37_I | 380.3 |
| 24 | 24.341 | Sebacic acid, octyl 2-propylpentyl ester | 78 | C_26_H_50_O_4_ | 426.6 |
| 25 | 24.478 | Octacosane | 97 | C_28_H_58_ | 394.7 |
| 26 | 25.172 | Dotriacontyl pentafluoropropionate | 83 | C_35_H_65_F_5_O_2_ | 612.8 |
| 27 | 25.404 | Dotriacontyl pentafluoropropionate | 91 | C_35_H_65_F_5_O_2_ | 612.8 |
| 28 | 25.898 | Octadecane, 1-iodo- | 95 | C_18_H_37_I | 380.3 |
| 29 | 25.919 | Dotriacontyl pentafluoropropionate | 80 | C_35_H_65_F_5_O_2_ | 612.8 |
| 30 | 26.162 | Tetrapentacontane, 1,54-dibromo- | 92 | Not found in the database | |
| 31 | 26.225 | Dotriacontyl pentafluoropropionate | 87 | C_35_H_65_F_5_O_2_ | 612.8 |
| 32 | 26.709 | Tetracosane | 95 | C_24_H_50_ | 338.6 |
| 33 | 26.772 | Eicosane, 2-cyclohexyl- | 64 | C_26_H_52_ | 364.6 |
| 34 | 27.024 | Octatriacontyl pentafluoropropionate | 93 | C_41_H_77_F_5_O_2_ | 697 |
| 35 | 27.098 | Dotriacontyl pentafluoropropionate | 87 | C_41_H_77_F_5_O_2_ | 697 |
| 36 | 27.645 | Tetracosane | 95 | C_24_H_50_ | 338.6 |
|  |  |  |  |  |  |

**Table.S3:** GC-MS identified components of the crude extract of *V. dahliae* at pH7. (Volatile compounds are listed in ascending order of Retention Time).

| **NO** | **Retention Time (min)** | **Compounds** | **Percentage**  **Match %** | **Molecular formula** | **Molecular Weight** |
| --- | --- | --- | --- | --- | --- |
| 1 | 3.213 | Acetic acid, butyl ester | 83 | C_6_H_12_O_2_ | 116.1 |
| 2 | 3.802 | Ethylbenzene | 91 | C_8_H_10_ | 106.1 |
| 3 | 3.918 | p-Xylene | 97 | C_8_H_10_ | 106.1 |
| 4 | 4.181 | Benzene, 1,3-dimethyl- | 97 | C_8_H_10_ | 106.1 |
| 5 | 8.832 | Citronellol | 98 | C_10_H_20_O | 156.2 |
| 6 | 11.01 | Methyleugenol | 98 | C_11_H_14_O_2_ | 178.2 |
| 7 | 11.547 | Dimethyl phthalate | 97 | C_16_H_22_O_4_ | 278.3 |
| 8 | 17.302 | Dibutyl phthalate | 94 | C_16_H_22_O_4_ | 278.3 |
| 9 | 17.449 | n-Hexadecanoic acid | 99 | C_16_H_32_O_2_ | 256.4 |
| 10 | 18.028 | Eicosane | 99 | C_20_H_42_ | 282.5 |
| 11 | 18.954 | Heneicosane | 99 | C_21_H_44_ | 296.5 |
| 12 | 19.848 | Heptadecane | 95 | C_17_H_36_ | 394.7 |
| 13 | 20.711 | Heptadecane | 97 | C_17_H_36_ | 394.7 |
| 14 | 21.269 | Hexanedioic acid, bis(2-ethylhexyl) ester | 95 | C_22_H_42_O_4_ | 370.5 |
| 15 | 22.311 | Pentacosane | 99 | C_25_H_52_ | 352.6 |
| 16 | 22.384 | Bis(2-ethylhexyl) phthalate | 98 | C_24_H_38_O_4_ | 390.5 |
| 17 | 23.047 | Tetracosane | 97 | C_24_H_50_ | 338.6 |
| 18 | 23.773 | Tetracosane | 96 | C_24_H_50_ | 338.6 |
| 19 | 24.341 | Sebacic acid, octyl 2-propylpentyl ester | 83 | C_26_H_50_O_4_ | 426.6 |
| 20 | 24.478 | Octacosane | 99 | C_28_H_58_ | 394.7 |
| 21 | 24.583 | Squalene | 99 | C_30_H_50_ | 410.7 |
| 22 | 24.731 | Octacosane | 59 | C_28_H_58_ | 394.7 |
| 23 | 25.162 | Fumaric acid, 2-chloroethyl pentadecyl ester | 91 | Not found in the database | |
| 24 | 25.899 | Triacontane | 94 | C_30_H_62_ | 422.8 |
| 25 | 26.162 | Octadecane, 1-iodo- | 94 | C_18_H_37_I | 338.6 |
| 26 | 26.214 | Dotriacontyl pentafluoropropionate | 87 | C_41_H_77_F_5_O_2_ | 612.8 |
| 27 | 26.709 | Carbonic acid, eicosyl vinyl ester | 91 | C_23_H_44_O_3_ | 368.5 |
| 28 | 27.014 | Octatriacontyl pentafluoropropionate | 93 | C_41_H_77_F_5_O_2_ | 697.0 |
| 29 | 27.635 | Octadecane | 86 | C_28_H_58_ | 394.7 |
| 30 | 28.003 | Hexadecane, 1-iodo- | 93 | C_16_H_33_I | 352.3 |
| 31 | 28.729 | 1-Hexacosene | 83 | C_26_H_52_ | 364.6 |
| 32 | 29.108 | Tetracosane | 90 | C_24_H_50_ | 338.6 |

**Table.S4:** GC-MS identified components of the crude extract of XEGI50 at pH3. (Volatile compounds are listed in ascending order of Retention Time).

| **NO** | **Retention Time (min)** | **Compounds** | **Percentage**  **Match %** | **Molecular formula** | **Molecular Weight** |
| --- | --- | --- | --- | --- | --- |
| 1 | 3.213 | Acetic acid, butyl ester | 83 | C_6_H_12_O_2_ | 116.1 |
| 2 | 3.803 | Ethylbenzene | 91 | C_8_H_10_ | 106.1 |
| 3 | 3.908 | p-Xylene | 97 | C_8_H_10_ | 106.1 |
| 4 | 4.181 | o-Xylene | 95 | C_8_H_10_ | 106.1 |
| 5 | 11.547 | Dimethyl phthalate | 92 | C_16_H_22_O_4_ | 278.3 |
| 6 | 17.281 | Dibutyl phthalate | 96 | C_16_H_22_O_4_ | 278.3 |
| 7 | 18.018 | Eicosane | 99 | C_20_H_42_ | 282.5 |
| 8 | 18.954 | Heneicosane | 98 | C_21_H_44_ | 296.5 |
| 9 | 19.838 | Heptadecane | 96 | C_28_H_58_ | 394.7 |
| 10 | 20.701 | Octadecane | 97 | C_28_H_58_ | 394.7 |
| 11 | 21.269 | Hexanedioic acid, bis(2-ethylhexyl) ester | 93 | C_22_H_42_O_4_ | 370.5 |
| 12 | 21.511 | Tetracosane | 99 | C_24_H_50_ | 338.6 |
| 13 | 22.3 | Pentacosane | 98 | C_25_H_52_ | 352.6 |
| 14 | 22.374 | Phthalic acid, di(2-propylpentyl) ester | 91 | C_24_H_38_O_4_ | 390.5 |
| 15 | 23.047 | Tetracosane | 96 | C_24_H_50_ | 338.6 |
| 16 | 24.478 | Octacosane | 96 | C_28_H_58_ | 394.7 |
| 17 | 25.162 | Ethanol, 2-(octadecyloxy)- | 83 | C_20_H_42_O_2_ | 314.5 |

**Table. S5:** GC-MS identified components of the antibiosis crude extract of XEGI50 and *V. dahliae* mixture at pH7. (Volatile compounds are listed in ascending order of Retention Time).

| **NO** | **Retention Time (min)** | **Compounds** | **Percentage**  **Match %** | **Molecular formula** | **Molecular Weight** |
| --- | --- | --- | --- | --- | --- |
| 1 | 3.813 | Ethylbenzene | 81 | C_8_H_10_ | 106.16 |
| 2 | 3.929 | p-Xylene | 97 | C_8_H_10_ | 106.17 |
| 3 | 4.107 | Butanoic acid, 2-methyl- | 83 | C_5_H_10_O2 | 102.13 |
| 4 | 4.192 | p-Xylene | 95 | C_8_H_10_ | 106.16 |
| 5 | 6.086 | Butanoic acid, 2-hydroxy-3-methyl- | 83 | C_5_H_10_O_3_ | 118.13 |
| 6 | 8.832 | Citronellol | 96 | C_10_H_20_O | 156.26 |
| 7 | 9.032 | Benzeneacetic acid | 94 | C_8_H_8_O_2_ | 136.15 |
| 8 | 9.747 | Naphthalene, 1-methyl- | 96 | C_11_H_10_ | 142.2 |
| 9 | 11.568 | Dimethyl phthalate | 94 | C_10_H_10_O_4_ | 194.18 |
| 10 | 16.786 | Di-sec-butyl phthalate | 93 | C_16_H_22_O_4_ | 278.34 |
| 11 | 17.155 | Hexadecanoic acid, methyl ester | 99 | C_17_H_34_O_2_ | 270.45 |
| 12 | 17.397 | 1,2-Benzenedicarboxylic acid, bis(2-methylpropyl) ester | 90 | C_16_H_22_O_4_ | 278.34 |
| 13 | 17.46 | n-Hexadecanoic acid | 99 | C_16_H_32_O_2_ | 256.42 |
| 14 | 18.028 | Eicosane | 99 | C_20_H_42_ | 282.54 |
| 15 | 18.712 | 9,12-Octadecadienoic acid (Z,Z)-, methyl ester | 99 | C_19_H_34_O_2_ | 294.47 |
| 16 | 18.796 | 9-Octadecenoic acid, methyl ester, (E)- | 99 | C_19_H_36_O_2_ | 296.48 |
| 17 | 18.954 | Heneicosane | 99 | C_21_H_44_ | 296 |
| 18 | 19.038 | Heptadecanoic acid, 16-methyl-, methyl ester | 99 | C_19_H_38_O_2_ | 298.5 |
| 19 | 19.312 | Octadecanoic acid | 99 | C_18_H_36_O_2_ | 284.48 |
| 20 | 19.848 | Eicosane | 98 | C_20_H_42_ | 268 |
| 21 | 20.017 | 1,2-Benzenedicarboxylic acid, butyl 2-ethylhexyl ester | 91 | C_20_H_30_O_4_ | 334.45 |
| 22 | 20.711 | Heptadecane | 97 | C_17_H_36_ | 240.46 |
| 23 | 21.279 | Hexanedioic acid, dioctyl ester | 95 | C_22_H_42_O_4_ | 370.57 |
| 24 | 21.521 | Tetracosane | 99 | C_19_H_40_ | 268 |
| 25 | 22.016 | 2-Methyltetracosane | 92 | C_25_H_52_ | 352.6 |
| 26 | 22.426 | Bis(2-ethylhexyl) phthalate | 97 | C_24_H_38_O_4_ | 390.5 |
| 27 | 23.047 | Octadecane | 97 | C_18_H_38_ | 254.4 |
| 28 | 23.773 | Hexadecane, 1-iodo- | 93 | C_16_H_33_I | 352.3 |
| 29 | 24.352 | Decanedioic acid, bis(2-ethylhexyl) ester | 95 | C_26_H_50_O_4_ | 426.6 |
| 30 | 24.478 | Octacosane | 97 | C_28_H_58_ | 394.71 |
| 31 | 24.741 | 1-Hexacosene | 84 | C_26_H_52_ | 364.6 |
| 32 | 25.172 | Eicosane, 9-cyclohexyl- | 87 | C_26_H_52_ | 364.6 |
| 33 | 25.92 | Triacontyl heptafluorobutyrate | 59 | C_34_H_61_F_7_O_2_ | 634.8 |
| 34 | 26.151 | Tetrapentacontane, 1,54-dibromo- | 91 | C_54_H_108_Br_2_ | 834 |
| 35 | 27.014 | Tetracosane | 94 | C_24_H_50_ | 338.6 |
| 36 | 27.992 | Dotriacontyl heptafluorobutyrate | 91 | C_36_H_65_F_7_O_2_ | 662.8 |
| 37 | 29.118 | Tetracosane | 90 | C_24_H_50_ | 338.6 |

**Table.S6:** **GC-MS identified components of the antibiosis crude extract of XEGI50 and** ***V. dahliae* mixture at pH3. (Volatile Compounds are listed in ascending order of Retention Time).**

| **NO** | **Retention Time (min)** | **Compounds** | **Percentage**  **Match %** | **Molecular formula** | **Molecular Weight** |
| --- | --- | --- | --- | --- | --- |
| 1 | 3.803 | Ethylbenzene | 91 | C_8_H_10_ | 106.1 |
| 2 | 3.918 | p-Xylene | 97 | C_8_H_10_ | 106.1 |
| 3 | 4.192 | o-Xylene | 94 | C_8_H_10_ | 106.1 |
| 4 | 4.392 | Butanoic acid, 2-methyl- | 83 | C_5_H_10_O_2_ | 102.1 |
| 6 | 5.17 | Pentanoic acid, 4-methyl- | 86 | C_6_H_12_O_2_ | 116.1 |
| 7 | 5.802 | 2(3H)-Furanone, dihydro-3-hydroxy-4,4-dimethyl-, (.+/-.)- | 91 | C_6_H_10_O_3_ | 130.1 |
| 8 | 6.233 | Butanoic acid, 2-hydroxy-3-methyl- | 83 | C_5_H_10_O_3_ | 118.1 |
| 9 | 8.843 | Citronellol | 98 | C_10_H_20_O | 156.2 |
| 10 | 8.927 | Picolinamide | 80 | C_6_H_6_N_2_O | 122.1 |
| 11 | 9.169 | Benzeneacetic acid | 94 | C_8_H_8_O_2_ | 136.1 |
| 12 | 9.758 | Naphthalene, 2-methyl- | 96 | C11H10 | 142.2 |
| 13 | 10.737 | n-Decanoic acid | 95 | C_10_H_20_O_2_ | 172.2 |
| 14 | 11.578 | Dimethyl phthalate | 93 | C_10_H_10_O_4_ | 194.1 |
| 15 | 12.894 | Benzeneacetic acid, 4-hydroxy- | 90 | C_8_H_8_O_3_ | 152.1 |
| 16 | 16.787 | Di-sec-butyl phthalate | 90 | C_16_H_22_O_4_ | 278.3 |
| 17 | 17.397 | 1,2-Benzenedicarboxylic acid, bis(2-methylpropyl) ester | 90 | C_20_H_30_O_4_ | 334.4 |
| 18 | 17.471 | n-Hexadecanoic acid | 99 | C_16_H_32_O_2_ | 256.4 |
| 19 | 18.028 | Eicosane | 99 | C_20_H_42_ | 282.5 |
| 20 | 18.954 | Heneicosane | 99 | C_21_H_44_ | 296 |
| 21 | 19.322 | Octadecanoic acid | 99 | C_18_H_36_O_2_ | 284.4 |
| 22 | 19.849 | Heptadecane | 97 | C_17_H_36_ | 240.4 |
| 23 | 20.017 | 1,2-Benzenedicarboxylic acid, butyl 2-ethylhexyl ester | 91 | C_20_H_30_O_4_ | 334.4 |
| 24 | 20.701 | Heneicosane | 97 | C_21_H_44_ | 296.5 |
| 25 | 21.28 | Hexanedioic acid, bis(2-ethylhexyl) ester | 99 | C_22_H_42_O_4_ | 370.5 |
| 26 | 21.511 | Tetracosane | 99 | C_24_H_50_ | 338.6 |
| 27 | 22.006 | 2-Methyltetracosane | 59 | C_25_H_52_ | 352.6 |
| 28 | 22.437 | Bis(2-ethylhexyl) phthalate | 92 | C_24_H_38_O_4_ | 390.5 |
| 29 | 23.047 | Heneicosane | 97 | C_21_H_44_ | 296.5 |
| 30 | 24.341 | Decanedioic acid, bis(2-ethylhexyl) ester | 95 | C_26_H_50_O_4_ | 426.6 |
| 31 | 24.468 | 1-Bromodocosane | 87 | C_22_H_45_Br | 389.4 |
| 32 | 25.162 | Eicosane, 9-cyclohexyl- | 87 | C_26_H_52_ | 364.6 |
